# Arbekacin treatment of a patient infected with a *Pseudomonas putida* producing a metallo-beta-lactamase

**DOI:** 10.1186/2052-0492-1-3

**Published:** 2013-10-23

**Authors:** Yoshiaki Iwashita, Tomoyuki Enokiya, Kei Suzuki, Kazuto Yokoyama, Akitaka Yamamoto, Ken Ishikura, Masahiro Okuda, Hiroshi Imai

**Affiliations:** Emergency and Critical Care Center, Mie University Hospital, Edobashi 2-174, Tsu, Mie, 514-8507 Japan

**Keywords:** Metallo-beta-lactamase, *Pseudomonas putida*, Arbekacin

## Abstract

Treatment of infections caused by multidrug-resistant *Pseudomonas* species is difficult because few antibiotics active against such organisms are available. Arbekacin, a relatively new aminoglycoside, is effective against *Pseudomonas* spp. *in vitro*. However, no clinical report on arbekacin treatment of a human infection with a multidrug-resistant *Pseudomonas* has appeared to date. We encountered a case of pneumonia caused by a *Pseudomonas* strain producing a metallo-beta-lactamase; the patient was successfully treated with arbekacin. A 69-year-old male presented to our hospital experiencing cardiac arrest after rescue from water. Spontaneous circulation had earlier resumed after brief application of cardiopulmonary resuscitation. The patient was subjected to induced hypothermia. He experienced severe acute respiratory distress syndrome. The patient regained consciousness on day 8 post-admission. Episodes of ventilator-associated pneumonia were recorded on days 5 and 12. The causative organism was a strain of *Pseudomonas putida* that produced a metallo-beta-lactamase. Combination therapy with arbekacin and levofloxacin successfully resolved the pneumonia. The patient was transferred to another hospital on day 37 to undergo further rehabilitation. Strains of *P. putida* producing metallo-beta-lactamases have become more widespread in recent years. Colistin is traditionally the drug of last resort to treat infections with multidrug-resistant *Pseudomonas*. However, colistin use is associated with a very high frequency of adverse effects, and the costs of such therapy are not covered by the Japanese health insurance system. Our results indicate that arbekacin is an efficient alternative to multidrug-resistant *Pseudomonas*.

## Background

Infections caused by *Pseudomonas putida* strains that produce metallo-beta-lactamases (MBLs) are difficult to treat because effective antibiotics are lacking. Colistin, the traditional drug of last resort, is not available in Japan, and the choice of antibiotic treatment for *P. putida* infections is thus limited. Combination therapy including arbekacin (ABK), an aminoglycoside antibiotic, has been reported to be effective against multidrug-resistant *Pseudomonas aeruginosa in vitro*[1]. To the best of our knowledge, no clinical report on ABK treatment of pneumonia caused by MBL-producing *P. putida* has yet appeared. We present a case featuring successful treatment, using ABK, of pneumonia caused by MBL-producing *P. putida*.

## Case presentation

A 69-year-old male presented to our hospital post cardiac arrest. The patient had been in a fishing boat that had accidentally overturned. The patient was rescued by others nearby, who called an ambulance. When the paramedics arrived on scene, his Glasgow Coma Scale was 1-1-1, and his SpO_2_ is 76% (10 L/min reservoir mask). Cardiac arrest occurred in the ambulance. Spontaneous circulation returned after 2 min of CPR. He was intubated on scene and was transferred to our hospital by helicopter.

**Figure 1:**
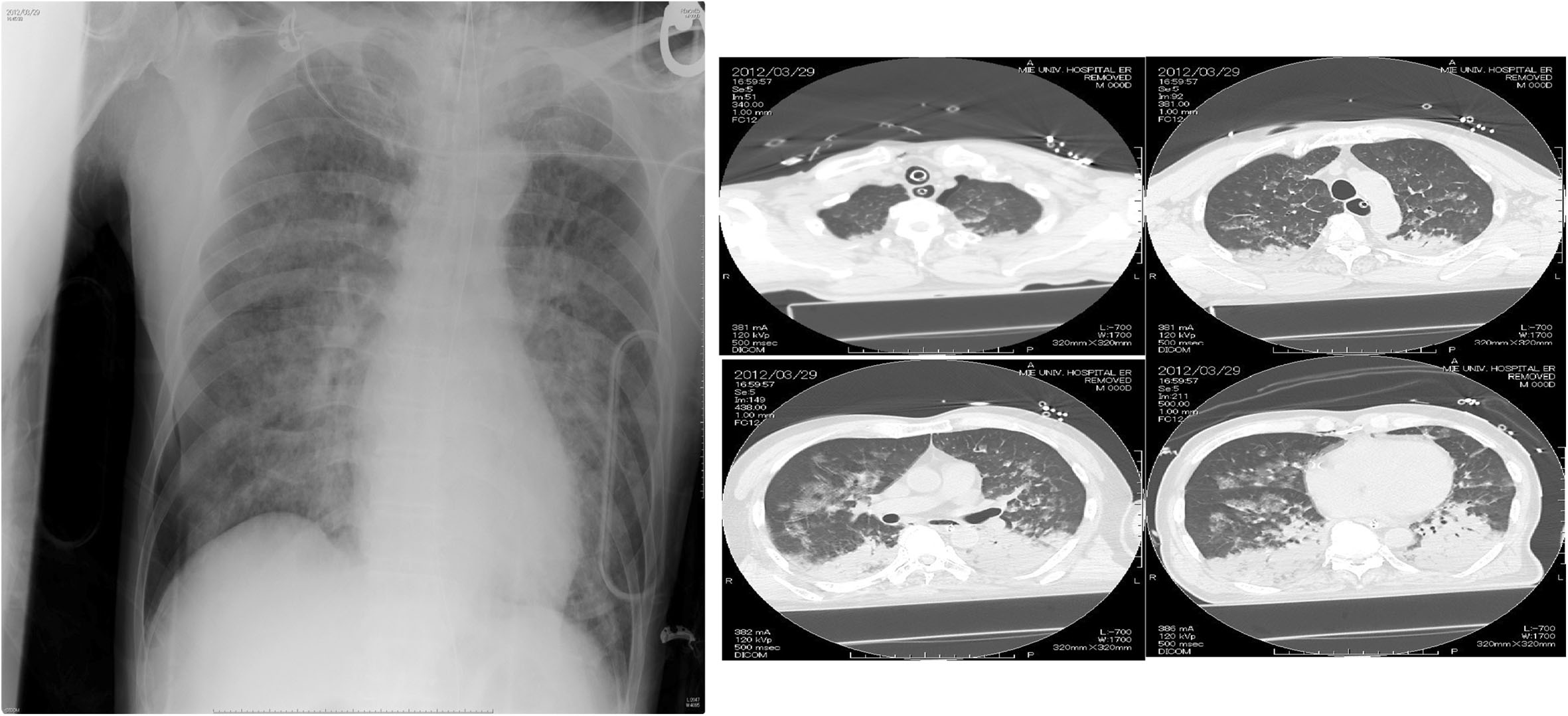
**Chest X-ray and CT scan upon ICU admission.** Chest radiograph shows diffuse infiltrate bilaterally.

On arrival at our hospital, his vital signs were BP 122/60 mmHg, HR 71 beats/min, and SpO_2_ 93% (SIMV FiO_2_ 1.0, PEEP 10 cmH_2_O, peak inspiratory pressure 25 cmH_2_O, and pressure support 10 cmH_2_O). The Glasgow Coma Scale score was 1-T-5, and the diameter of his pupils was 4 mm × 4 mm with light reflex of +/+. Respiratory sounds were evident as coarse crackles bilaterally. A chest X-ray showed diffuse bilateral infiltration (Figure 1). A chest CT scan revealed infiltrates bilaterally in the dependent lung regions and left multiple costal fractures with pneumohemothorax, probably attributable to chest compression. Arterial blood gas revealed the presence of mixed acidosis with severe hypoxia: pH 7.04, PaO_2_ 52 mmHg, PaCO_2_ 52 mmHg, HCO_3_ 14.1 mmol/L, BE −16.6 mmol/L, and lactate 5.4 mmol/L. The white blood cell count was slightly elevated to 12,470/μL (Table 1).

**Table 1:** **Laboratory data on arrival**

	Number	Unit
WBC	12,470	/μL
Hb	12.6	g/dL
Platelet	231,000	/μL
Total protein	6.9	g/dL
Albumin	4.0	g/dL
Total bilirubin	0.5	mg/dL
AST	74	IU/L
ALT	47	IU/L
LDH	450	IU/L
ALP	259	IU/L
CRP	0.1	mg/dL
CK	679	IU/L
BUN	26	mg/dL
Creatinine	1.25	mg/dL
Na	146	mmol/L
K	3.4	mmol/L
Cl	113	mmol/L
PT-INR	0.82	
APTT	16.2	sec
pH	7.04	
PCO2	52.0	mmHg
PO2	85.0	mmHg
HCO3	14.1	mmol/L
BE	−16.6	mmol/L
Lactate	5.4	mmol/L

Metabolic acidosis with lactate elevation was seen.

**Figure 2:**
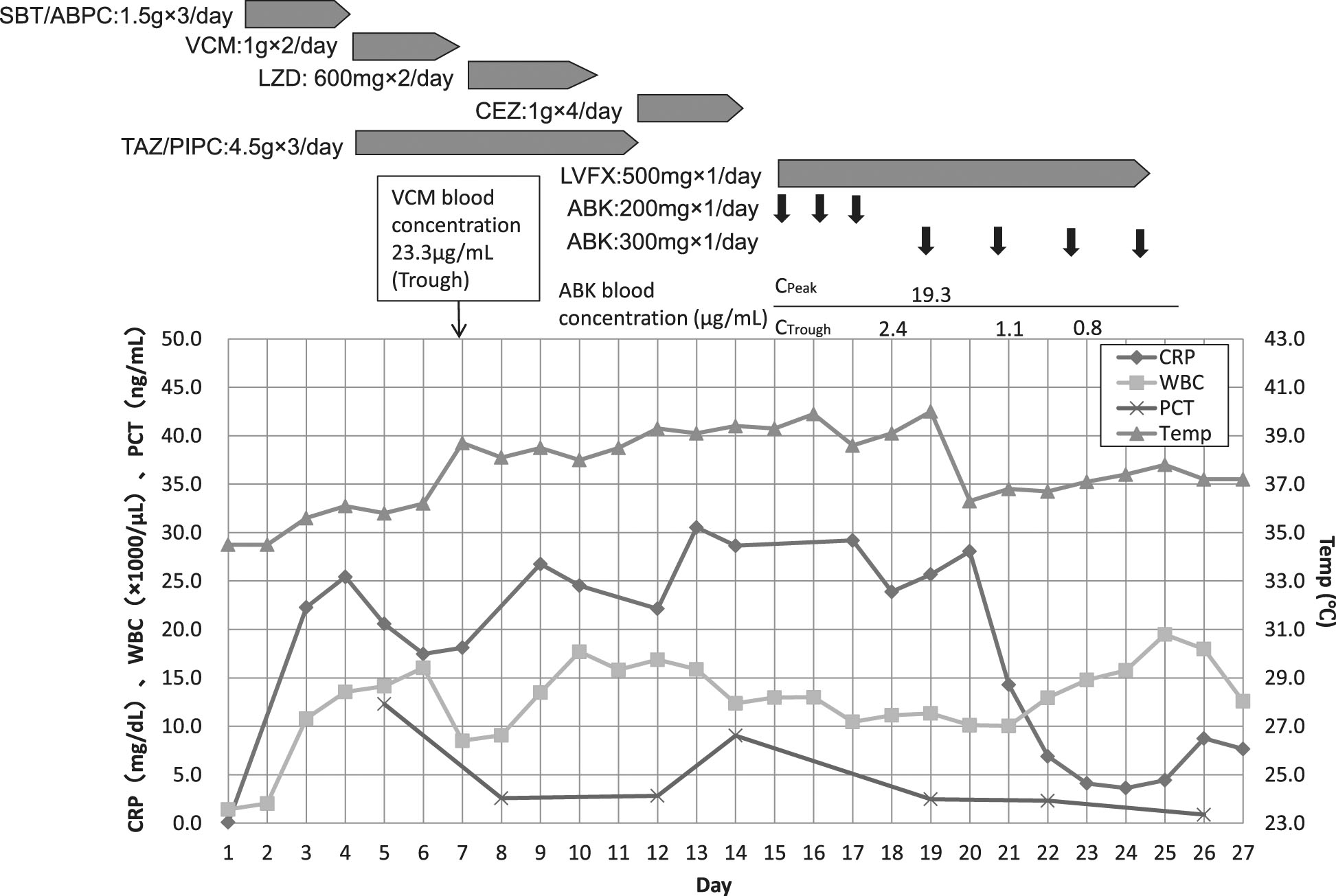
**Clinical course of the patient.** Inflammatory markers, dose, and duration of the antibiotics used are shown.

The patient was diagnosed as post cardiac arrest due to severe hypoxia. He was subjected to induced hypothermia (34°C for 24 h). A chest drainage tube was inserted to treat the left-side pneumohemothorax. The ventilator setting was changed to the airway pressure-releasing ventilation mode; the relevant settings were *P*_high_ 26 cmH_2_O, *P*_low_ 5 cmH_2_O, *T*_high_ 3.5 s, *T*_low_ 0.5 s, and FiO_2_ 0.6. The ARDS was treated via nitric oxide inhalation (10 ppm), and the patient was placed in prone position. Sulbactam-ampicillin (1.5 g every 6 h) treatment was commenced for prophylaxis of aspiration pneumonia. The clinical course is shown in Figure 2. The patient was rewarmed on day 6, at which time he had fully recovered neurologically.

On day 4, the patient developed late-onset ventilator-associated pneumonia. We initiated empirical therapy with tazobactam-piperacillin 4.5 g every 8 h and vancomycin hydrochloride 1 g every 12 h. The patient experienced general edema and impairment of renal function, and the blood concentration of vancomycin hydrochloride was thus much greater than the predicted value. We, therefore, replaced this drug with linezolid on day 7. Culture of sputum taken on day 7 revealed the presence of methicillin-sensitive *Staphylococcus aureus*. We changed linezolid to cefazolin on day 11. Although the inflammatory symptoms improved slightly, fever and sputum production became worse on day 12, and we suspected that a second episode of ventilator-associated pneumonia had developed. The patient required tracheostomy on day 13. On day 15, sputum culture taken on day 12 revealed the presence of a *P. putida* strain that produced an MBL (to be of the integron-encoded type revealed on day 38). The method of *P. putida* isolation was semi-quantitative, and the method of MBL detection was PCR method. Of the antibiotics tested, the bacterium was sensitive only to amikacin (AMK), arbekacin, and minocycline. The minimum inhibitory concentration of ABK was 2.0 μg/mL. The blood concentrations of AMK could not be measured in our hospital, whereas that of ABK can. We, therefore, chose to employ this drug in treatment. As reported above, the blood concentration of vancomycin exceeded the predicted value when the drug was used in the earlier treatment. We, therefore, resolved to carefully titrate ABK levels in the blood. Our final choice of therapy was ABK 300 mg every 24 h with levofloxacin (LVFX) 500 mg every 24 h. We titrated ABK doses to maintain *C*_peak_ values over 16 μg/mL and *C*_trough_ values less than 2.0 μg/mL. Inflammatory symptoms had decreased in extent by day 17, but treatment with both drugs was continued up to day 26. The patient was transferred to another hospital on day 37 for further rehabilitation.

### Discussion

The increasing prevalence of drug-resistant microbes is a major issue in modern critical care. Infections caused by MBL-producing *P. putida* are difficult to treat, especially in Japan, where health insurance covers the use of relatively few antibiotics. Here, we report on a case of pneumonia caused by MBL-producing *P. putida*; the patient was successfully treated with ABK.

*P. putida* is a fluorescent member of the *Pseudomonas* family and causes nosocomial infections, including pneumonia, urinary tract infections, neonatal infections, and adult bacteremia [2, 3]. Pneumonia caused by *P. putida* is suspected to be associated with high mortality rates when bacteremia develops [3, 4]. Our patient had experienced cardiac arrest and was subsequently subjected to induced hypothermia to prevent hypoxic brain injury. Our patient was, thus, susceptible to opportunistic nosocomial infections, especially of the lung.

Historically, *P. putida* has been considered to be an opportunistic pathogen of low virulence and has been susceptible to a range of antibiotics [5]. However, in both Japan and Korea, the isolation frequencies of imipenem-resistant *Pseudomonas* spp. (especially *P. aeruginosa* and *P. putida*) are increasing [6]. Most imipenem-resistant *P. aeruginosa* strains produce MBLs. In Korea, 8 of 12 (67%) imipenem-resistant *P. putida* isolated in 2005 expressed MBLs [6]. The MBL element of *P. putida* can be horizontally transferred to *P. aeruginosa*[7]. To date, eight types of MBLs have been identified and are known by the acronyms IMP, VIM, SPM, SIM, GIM, DIM, AIM, and KHM [8]. The IMP type was identified in the strain that infected our patient; this is the most common form of MBL in Japan. MBL detection was done by PCR method [9].

Colistin is the antibiotic of choice for treating infections with MBL-producing *Pseudomonas* spp. [10]. However, colistin therapy is not supported by Japanese health insurance. Arbekacin is a broad-spectrum aminoglycoside antibiotic, effective in treating infections with a range of bacteria, from Gram-positive cocci to Gram-negative bacilli. This antibiotic is indicated for treatment of infections with methicillin-resistant *S. aureus* and has been reported to effectively kill *Pseudomonas* spp. *in vitro*. However, the clinical effectiveness of ABK is not well known. Use of aminoglycoside antibiotics is associated with development of renal toxicity; the blood levels of such drugs must be therapeutically monitored. We initially prescribed VCM for our patient but found that the blood concentration thereof was much higher than what was expected and threatened to compromise renal function. We, thus, resolved to carefully monitor aminoglycoside levels in the blood of our patient. Of the drugs to which the isolate was sensitive, we chose to prescribe the aminoglycoside ABK rather than AMK because it was possible to measure ABK blood concentrations (but not those of AMK) in our institution. ABK is a relatively new drug, and the pharmacokinetics and pharmacodynamics thereof remain not to be fully investigated. The optimal blood concentration of ABK has not yet been established. For aminoglycoside drugs, the ratio of peak blood concentration (*C*_peak_) to the minimal inhibitory concentration has been reported to be related to the clinical efficacy. Thus, a *C*_peak_/MIC value greater than 8 was associated with a clinical efficacy of over 90% [11]. We therefore maintained the *C*_peak_ level of ABK over 16 μg/mL and the *C*_trough_ level below 2.0 μg/mL. We found that we could successfully maintain the desired drug blood concentrations by varying the drug dose.

We choose to additionally prescribe LVFX for two reasons. First, our patient had not earlier received any quinolone. Second, *P. putida* that are resistant to fluoroquinolones express a drug efflux system [12, 13]. We therefore considered that LVFX might nonetheless be effective if given at high concentrations. However, later laboratory analysis revealed that the combination of ABK and LVFX was no more effective against the isolate *in vitro* than was ABK alone. Therefore, the observed clinical effect may have been attributable to the action of ABK alone.

The clinical course of our patient was favorable; no adverse events were noted. Thus, we propose that combination therapy that includes ABK may effectively treat infections with MBL-producing *Pseudomonas* spp.

## Conclusion

We experienced a case of MBL-producing *P. putida* successfully treated with ABK. ABK is an effective choice of treatment for MBL-producing *Pseudomonas* spp.

## Consent

Written informed consent was obtained from the patient for publication of this case report and any accompanying images. A copy of the written consent is available for review by the Editor-in-Chief of this journal.
